# Peritoneal Adhesions. Appendicitis. Fracture of Humerus. Septicemia1Notes from a clinic at the New York Post Graduate Medical School, December 2, 1904.

**Published:** 1905-01

**Authors:** Robert T. Morris

**Affiliations:** New York


### CLINICAL REPORTS.

### Peritoneal Adhesions. Appendicitis. Fracture of Humerus. Septicemia.[1]

By ROBERT T. MORRIS, M. D., New York.

PERITONEAL ADHESIONS. PREVENTION OF RECURRENCE.

CASE I.—Four years ago this young woman was operated upon for appendicitis. I judge from her description that the case was one with abscess. Since her recovery from the acute disturbance, she has suffered from persistent pain and occasional attacks of tenderness in the abdominal region. This is characteristic of the disturbance caused by peritoneal adhesions. There are other features belonging to the condition, for example, intestinal fermentation, dysmenorrhea, and spinal irritation, but if we make too elaborate a classification of symptoms we are apt to speculate upon a more elaborate diagnosis, and to let slip the bull's eye symptoms that are to be held up as the target for our efforts—namely, pain in the abdomen of persistent character, and occasional attacks of abdominal tenderness, following an attack of peritonitis.

Was the operation in any way responsible for the chronic invalidism? That we cannot say, for we do not know whether gauze packing was employed or not. Inflammatory destruction of peritoneal endothelium is sufficient to cause chronic adhesion disturbances. The method of using gauze packing which was

1. Notes from a clinic at the New York Post Graduate Medical School, December 2, 1904.

in vogue for some years, and which is still used by some surgeons in septic abdominal work, is also responsible for many abdominal adhesions, because the gauze as a foreign body causes the peritoneum to throw out much protecting lymph.

The abdomen of this patient is now opened. Adhesions bind the omentum to the uterus and to loops of bowel. There are adhesion bands between various loops of bowel. We find the stump of the appendix.

Adhesion surfaces are all separated from each other. How can we prevent reformation of adhesions? By introducing a mechanical obstacle to such readhesion. The method which we employ for this case consists in applying strips of Cargile membrane to the separated adhesion surfaces. This material is the sterilised peritoneum from the ox. It is absorbed like catgut. Meanwhile, new endothelium will have shot across the bared surfaces, and we shall have glistening peritoneum where formerly there was connective tissue adhesion.

There is a good deal of oozing from the pelvis at the site of torn adhesions. Shall we enlarge the incision and make a tedious hunt for bleeding points? That would prolong the operation, and introduce a feature of surgical severity. Shall we put in a drain to carry the blood out of the abdomen? The patient might bleed to a danger point. We will close the abdomen. Blood in the peritoneal cavity is still in circulation, because the peritoneal cavity is a lymph chamber. Lymphatics all empty into veins, and this blood, or its serum at least, will continue its round in the vascular system. Beside that, atmospheric pressure will have a tendency to lessen the oozing as soon as the abdomen is closed. If arteries were spouting it would not be safe to follow this plan of procedure, but we know that the oozing is from torn veins. In ruptured extrauterine pregnancy many a patient lives with enormous blood losses into the peritoneal cavity, where death from hemorrhage would occur if the blood escaped externally.

### APPENDICITIS. PALPATION OF APPENDIX.

Case II.—This man had an attack of appendicitis in 1900 which kept him in bed for a month. He has had four subsequent attacks. Let us palpate the appendix before operation. Many physicians say that the appendix cannot be palpated under ordinary circumstances, and it is evident from observation of their efforts that they are perfectly honest in this belief. Let us try a special method. First, the ascending colon is allowed to slip out from beneath the fingers of the examining hand. That is a landmark. Follow it down to the cecum. Try to find the appendix at this point and failure will commonly result. Lift the cecum on the ends of the fingers by pressing deeply in the right direction. Still the appendix will remain hidden in many cases, although this movement brings out some appendices that cannot be felt otherwise. Now for the main trick; keep the examining fingers where they will lift the cecum, and then with the other hand placed on the other side of the abdomen press the viscera back and forth beneath the examining fingers. That allows us to feel minutely,—almost to see with the ends of the examining fingers.

This patient's appendix is in his pelvis. The part next the cecum is soft; then comes an inch of scar tissue, and beyond this there is a mucous inclusion. The appendix is adherent so that we cannot pull it up and measure it before the abdomen is opened. At the last clinic we described one appendix as three inches long, soft and pink. The house surgeon remarked that he had reached the point where he could feel gas in the appendix, but could not feel the color. The statement was made rather inadvertently about the color in that case, and merely described the sort of appendix that we expected to find, the abdomen being opened for another sort of condition.

In this case the abdomen is opened by the incision an inch and a half long. That is merely a good standard for length, and we can make an incision a foot long later if necessary. My argument is simply this: that it is better to let the patient off easily if we are able to do so. Through the short incision we now separate firm adhesions between the appendix and the iliac vessels. We may tear the iliac vein or artery. That may make no special difference to the patient excepting that we shall have to make a longer incision for suturing the torn vessels, and there is some special risk attending the procedure. The appendix is separated from the vessels, but the ureter now appears to be so firmly adherent that it cannot be separated without injury. It is necessary to enlarge the incision. The ureter is separated with some difficulty. The appendix is removed. The mucous inclusion at the tip contains a concretion.

### CLOSURE OF OVIDUCT.

Case III.—This young woman had pelvic inflammation after the birth of her last child, and has symptoms of peritoneal adhesions. We will treat those as in the first case today. Incidentally, we may repair the perineum which was torn. A horse-shoe incision is made in the perineal skin. The closed scissors are pushed into the tissues as far as the bulbo-cavernosus muscle on one side, and opened while in this position. That exposes the muscle. The step is repeated on the opposite side. The bulbo-cavernosus muscles are next approximated with catgut, and this brings together at the same time the remains of the levator ani muscles which were formerly united in the median raphé. The skin wound is sutured. Five or six minutes time will suffice for repairing most torn perineums by this method.

Adhesions about the adnexa are next separated through an abdominal incision. The patient is slight, weighing only eighty pounds. She has suffered much damage from the birth of her two children, and she and her husband do not wish to have similar damage repeated. What can we do that is morally right? Tie a silk thread about each oviduct near the fimbriated extremity. The ligature will become encapsulated, and will close the oviduct. If the patient decides later that she wishes more children we can easily make a small abdominal incision, and a simple slitting of the closed end of the oviduct will free it again for action.

COMPOUND FRACTURE OF THE HUMERUS. SERUM PROTECTION.

Case IV.—This boy has just been run over in the street, by a heavy wagon. His right humerus is fractured at several points, the muscles of the arm are crushed and full of extravasated blood. There is an external opening of the skin through which a fragment of bone probably protruded. How shall we treat the case? If we enlarge the opening for drainage, the whole crushed area is bound to become infected, and the boy is likely to lose his arm or his life. We will take a desperate chance, based on the idea that the blood serum from the opening has been sufficiently germicidal to keep the region of the aperture sterile. The region about the aperture is prepared aseptically and we close the opening with catgut. The fracture is now transformed from a compound one into a simple one, provided that asepsis can be maintained.

This method of treatment of compound fractures was one that some of us used at Bellevue Hospital before the days of antisepsis, and we have remembered the results that were then common, through all of the later revolution in methods. At that time we did not know the reason for obtaining primary union in such cases. Now we know about the germicidal action of blood serum while it is fresh. As soon as blood serum has become a culture medium in any given case, closure of the wound is likely to be disastrous in result. I would like to have the class follow the subsequent history in this boy's case, in which we have taken what seems to some a desperate chance, but in which we have simply gone back to one of the old-fashioned methods. As one gets older himself, his respect for many of the old-fashioned ideas increases.

SEPTICEMIA. BLOOD WASHING.

Case V.—The next patient is a young woman who had osteomyelitis of the tibia. Last week we followed the classical plan of removing the diaphysis of the tibia, and leaving the periosteum, which was expected to renew the tibia as perfectly as a severed newt's tail is renewed. The patient now has a temperature ranging above 105° F. Osteomyelitis is sometimes a purely streptococcus invasion of the cancellous structure of a long bone. Sometimes it is a purely bacillus tuberculosis invasion. Often it is a tuberculosis invasion, with streptococcus involvement as a terminal infection. The latter was apparently the case in the present instance, and I do not know whether the disturbance following the operation is due to septicemia, or to acute general tuberculosis. The blood examination at this early moment does not give us a decision in the matter, but something must be done. We shall wash toxins out of the blood. This is done by distending the bloodvessels with normal saline solution, so that the emunctories are called into quick action, and toxins are thrown out along with the excess of water. It is important in a case of this sort to remember that the nine-tenths of 1 per cent. solution is to be used. That is isotonic for human blood. The six-tenths of 1 per cent. salt solution is isotonic for frog's blood and might cause some solution of hemoglobin if used here.

We inject 2,000 cubic centimeters of the salt solution into one of the veins of the arm. In a few hours time this patient's temperature will probably have dropped several degrees. Blood washing not only removes toxins, but there is excited at the same time a protective hyperleucocytosis. Many a case of septicemia is instantly turned from one of grave danger into one of convalescence by a single injection of this sort, although it is necessary to remove the focus of infection to prevent recurrence of the toxemia as a rule.

In this case we cannot well remove the focus of infection without amputating the leg. That will be left for a last resort. An indication for amputation of the leg would be the development of a colliquative diarrhea. We may have to amputate for other evidences of a grave condition, but the development of colliquative diarrhea is commonly the signal for immediate action on our part when the question of amputation is on the tapis.

Note—Made after the lapse of twelve days—Primary union in cases I., III., and IV. Primary union except at small drain opening in case II. Symptoms of acute general tuberculosis became marked in Case V., and patient died from the meningeal feature.

616 Madison Avenue.

